# Supplementary material for: Measuring Impairments of Mentalization with the 15-Item Mentalization Questionnaire (MZQ) and Introducing the MZQ-6 Short Scale: Reliability, Validity and Norm Values Based on a Representative Sample of the German Population
Source: Diagnostics (Basel). 2022 Dec 30;13(1):135. doi: 10.3390/diagnostics13010135 (PMC9818984; doi:10.3390/diagnostics13010135)
Supplement: Supplementary file 1 [file diagnostics-13-00135-s001.zip › diagnostics-2040739-supplementary.pdf]

		4-Factor solution	3-Factor solution
No	Item		
1	If I expect to be criticized or offended, my fear increases more and more	Factor 1: Psychic equivalence mode	Factor 2: Mental states regarding oneself –affect-regulation
2	Explanations from others are of little assistance in understanding my feelings.	Factor 2: Affect regulation	Factor 1: Mental states in oneself regarding others–cognition and communication
3	Sometimes feelings are dangerous for me	Factor 2: Affect regulation	Factor 2: Mental states regarding oneself –affect-regulation
4	I only believe that someone really likes me a lot if I have enough realistic proof for it (e.g., a date, a gift or a hug).	Factor 1: Psychic equivalence mode	Factor 1: Mental states in oneself regarding others–cognition and communication
5	Most of the time it is better not to feel anything	Factor 3: Refusing self-reflection	Factor 3: Mental states regarding oneself –affect-perception and -differentiation
6	Often, I can't control my feelings	Factor 2: Affect regulation	Factor 2: Mental states regarding oneself –affect-regulation
7	It's difficult for me to believe that relationships can change.	Factor 1: Psychic equivalence mode	Factor 1: Mental states in oneself regarding others–cognition and communication
8	I tend to ignore feelings of physical tension or of discomfort until they compel my full attention	Factor 3: Emotional awareness	Factor 3: Mental states regarding oneself –affect-perception and -differentiation
9	Talking about feelings would mean that they become more and more powerful	Factor 3: Refusing self-reflection	Factor 1: Mental states in oneself regarding others–cognition and communication
10	Sometimes I only become aware of my feelings in retrospect	Factor 3: Emotional awareness	Factor 3: Mental states regarding oneself –affect-perception and -differentiation
11	Frequently it's difficult for me to perceive my feelings at their full intensity	Factor 3: Emotional awareness	Factor 3: Mental states regarding oneself –affect-perception and -differentiation
12	Often, I feel threatened by the idea that someone could criticize or offend me	Factor 1: Psychic equivalence mode	Factor 2: Mental states regarding oneself –affect-regulation
13	If someone yawns in my presence, that's a reliable sign that he is bored in my company	Factor 3: Refusing self-reflection	Factor 1: Mental states in oneself regarding others–cognition and communication
14	Most of the time I don't feel like talking about my thoughts and feelings with others	Factor 3: Refusing self-reflection	Factor 1: Mental states in oneself regarding others–cognition and communication
15	Often, I don't even know what is happening inside of me	Factor 3: Emotional awareness	Factor 3: Mental states regarding oneself –affect-perception and -differentiation
